# Hybrid procedure for treating adult congenital heart disease with valvular heart disease in two patients

**DOI:** 10.1186/s13019-019-1002-z

**Published:** 2019-10-23

**Authors:** Chun-sheng Li, Zhong Lu, Xiao-rong Song, Zhong-ya Yan

**Affiliations:** 1 Shandong University School of Medicine, Jinan, China; 2 Department of Cardiovascular Surgery, The Second Hospital of Anhui Medical University, Hefei, 230601 Anhui Province China; 3 Department of Cardiovascular Surgery, Anhui Provincial Hospital, Anhui Medical University, Hefei, China

**Keywords:** Adult congenital heart disease, Hybrid procedure, Valvular heart disease

## Abstract

**Background:**

The traditional approach for adult congenital heart disease combined with valvular disease is surgical treatment under cardiopulmonary bypass (CPB). This approach has a high incidence of postoperative complications, especially in patients with pulmonary hypertension and old age. We present two patients in whom the hybrid procedure was used to treat congenital malformations, followed by valve formation and replacement surgery.

**Case presentation:**

A 63-year-old man had a muscular ventricular septal defect complicated by mitral regurgitation and a 57-year-old man had patent ductus arteriosus complicated by aortic stenosis. In both of the patients, the congenital malformation was successfully treated by the hybrid procedure, followed by valve repair or replacement. Both patients had no complications. A post-procedure echocardiogram showed no residual shunt across the duct.

**Conclusions:**

Our findings suggest that the hybrid procedure is a useful alternative for treating adult congenital heart disease with valvular heart disease. This procedure reduces the surgical incision and difficulty of surgery, shortens the CPB time, avoids residual leakage after surgery, and reduces recovery and hospitalization times.

## Background

With advancement of medical technology, the incidence of congenital heart disease in adults has increased yearly, with more than 1.4 million adults with congenital heart disease in the USA alone [1]. Most of these patients have relatively simple heart disease, mostly due to a lack of screening in childhood in developing countries. These patients often visit a doctor because of a decrease in activity tolerance. Therefore, these patients have different degrees of pulmonary hypertension [2] or are combined with heart failure [3]. At present, most of these patients are treated with cardiac malformation and valve repair or replacement under CPB. However, longer CPB time, aortic cross clamp time and presence of pulmonary hypertension are associated with higher incidence of postoperative complications [4]. We report here two cases of adult congenital heart disease. One of these patients had a muscular ventricular septal defect (VSD) with mitral regurgitation and one had patent ductus arteriosus (PDA) and aortic stenosis. The hybrid procedure was used to treat the congenital malformations, followed by valve formation and replacement surgery in these two cases.

## Case presentation

### Patient 1

**Fig. 1:**
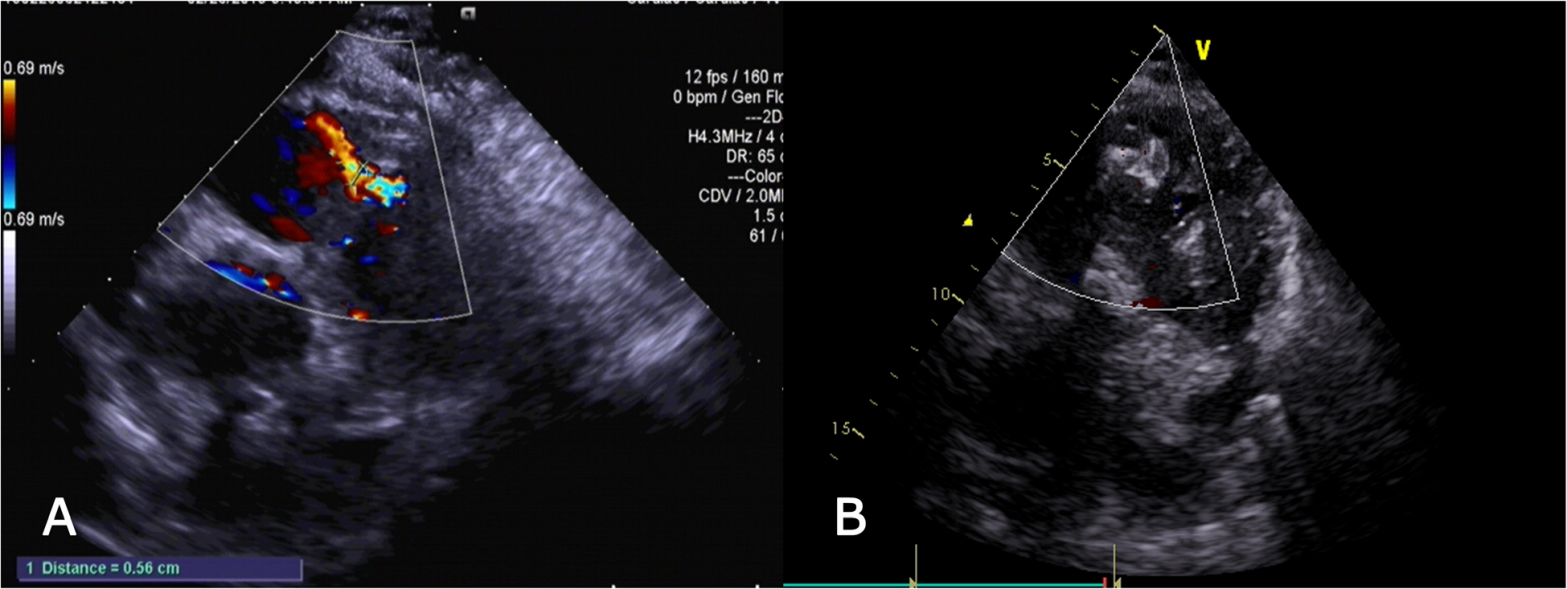
**a** Preoperative echocardiography shows muscular ventricular septal defect. **b** Postoperative echocardiography shows that the occluder is in a good position with no residual shunt

A 63-year-old man presented with dyspnea during exercise for longer than 1 month. A physical examination showed a systolic murmur of grade 3/6 at the left third to fifth intercostal spaces. Echocardiography showed an increase in the left ventricle, muscular VSD, and moderate mitral regurgitation. We decided to perform mitral valve repair after closure of the muscular VSD under direct vision with CPB. After median sternotomy was performed, we established CPB. After the heart stopped, the right atrium and the interatrial septum were cut. The left ventricular surface was examined to view the ventricular septal defect near the apex, and the diameter was approximately 6 mm. The P1214 PDA occluder (Starway Medical Technology, Inc., Beijing, China) was selected and the end was pre-stitched with 3–0 prolene suture. The occluder was placed into the left ventricle through the right ventricular surface, and the occluder was released. The pre-stitched 3–0 prolene suture was reinforced and stitched to the right ventricular surface (Fig. 1a, b). The mitral valve was then examined and underwent complete ring annuloplasty.

### Patient 2

**Fig. 2:**
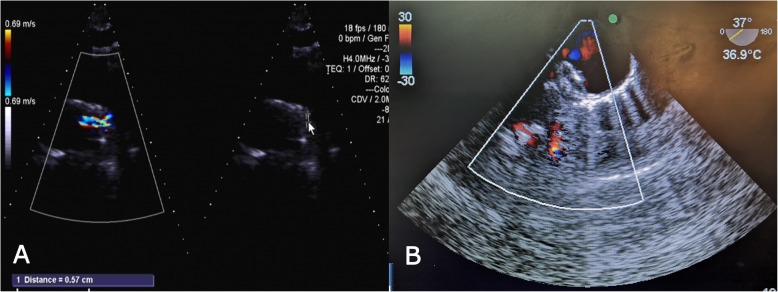
**a** Preoperative echocardiography shows patent ductus arteriosus. **b** Postoperative echocardiography shows that the occluder is in a good position with no residual shunt

A 57-year-old man presented with dyspnea during exercise for longer than 1 month. A physical examination showed a continuous murmur of grade 3/6 at the left second to third intercostal spaces. Echocardiography suggested an increase in the left ventricle, PDA (tubular), and moderate aortic regurgitation. We decided to first perform occlusion of the PDA by transesophageal echocardiography (TEE) under direct vision and then perform aortic valve replacement under CPB. After median sternotomy, we sutured a purse-string in the pulmonary artery. TEE of the PDA showed a diameter of approximately 6 mm and it was the tubular type. The P1214 PDA occluder was selected and the PDA was occluded through the pre-made purse-string of the pulmonary artery (Fig. 2a, b). After occlusion, CPB was established. Aortic valve replacement was performed after atrial arrest.

Both patients had no complications. A post-procedure echocardiogram showed no residual shunt across the duct. At 1 month of follow-up, the patients were well and there was no residual shunt in either patient.

## Discussion

The traditional approach for adult congenital heart disease with valvular disease is surgical treatment under CPB. Because of muscular VSD in the deep position in this condition, difficulty of suturing leads to a high probability of postoperative residual leakage. Some of these patients need to have larger left and right ventricular incisions [5]. In patients with PDA, pulmonary artery repair should be performed after the aorta is blocked. After an incision is made, exposing and suturing are difficult because of the high flow rate. The operation should even be stopped under deep hypothermia [6]. Therefore, open surgery of intracardiac malformations has gradually been replaced by interventional closure therapy for congenital heart disease [7, 8]. If congenital heart disease is combined with heart valvular disease and this requires hybrid procedure surgery, the VSD and PDA are blocked by transcatheter intervention before surgery. Valvular disease requires open thoracic surgery again, and the hospitalization time and cost are likely to be increased. Subsequent interventional mitral or aortic valve replacement can be performed in the catheter laboratory. However, because of the high rate of complications and high cost after interventional valve replacement, valve replacement is currently limited to older patients or patients with contraindications to open surgery. We performed the hybrid procedure for treating muscular VSD and PDA by standard median sternotomy. We then surgically performed mitral repair and aortic valve replacement to reduce the incision on the heart’s surface and shorten the recovery and hospitalization time. The advantage of the hybrid procedure is not only no requirement for a secondary surgery, but also a blocking operation under direct vision is simpler and more straightforward, without X-ray fluoroscopy, compared with interventional closure. In the case of VSD and PDA malformation, the advantages of the hybrid procedure can be achieved by a single surgical incision to reduce the incision of the heart surface, shorten the CPB time, and reduce the incidence of postoperative residual leakage compared with conventional repair.

## Conclusions

In the treatment of adult congenital heart disease with valvular heart disease, single-incision one-stop shop combined with the hybrid procedure with open surgery to treat such diseases appears to be a useful alternative. The advantages of this surgery compared with standard treatment are as follows. (1) The surgical incision is reduced, the operation is simpler, and the difficulty of surgery is greatly reduced. (2) The CPB time and aortic cross clamp time are shortened, and the myocardial protection effect is good. (3) Tissue damage is minimal to avoid residual leakage after surgery. (4) There is a fast recovery and shorter hospital stay. However, relatively high treatment costs may hinder implementation of this method.

## Data Availability

Not applicable.

## Abbreviations

CPB: Cardiopulmonary bypass
PDA: Patent ductus arteriosus
TEE: Transesophageal echocardiography
VSD: Ventricular septal defect
